# Renoprotective Effects of Total Glucosides from Paeony against Nephrotoxicity Induced by Total Alkaloids from* Semen Strychni*

**DOI:** 10.1155/2017/8256278

**Published:** 2017-10-22

**Authors:** Mingming Lv, Meiyu Zhang, Yezhe Cheng, Kexia Zhang, Chenzhi Hou, Xiaohui Chen

**Affiliations:** ^1^School of Pharmacy, Shenyang Pharmaceutical University, Shenyang 110016, China; ^2^School of Traditional Chinese Materia Medica, Shenyang Pharmaceutical University, Shenyang 110016, China

## Abstract

*Semen Strychni* have been shown to have therapeutic effect in improving blood circulation, relieving rheumatic pain, and treating cancer. However,* Semen Strychni* could cause severe nephrotoxicity. The present study was designed to evaluate whether treatment with total glucosides from paeony (TGP) has renoprotective effect against nephrotoxicity induced by total alkaloids from* Semen Strychni* (TAS). The levels of blood urea nitrogen (BUN) and creatinine (Cr) were determined and histopathological changes were also examined to evaluate renal injury. Moreover, a HPLC-MS method was developed and validated to investigate the comparative toxicokinetics of strychnine and brucine in rats plasma after oral administration of TAS and pretreatment with TGP. Results demonstrated that the levels of BUN and Cr were significantly increased (*p* < 0.05) in TAS group, together with tubule epithelium cloudy swelling, degeneration, and glomerular atrophy in rats' kidneys. The TAS-induced kidney damage was alleviated after pretreatment with TGP. Besides, *T*_max_ of strychnine and brucine were increased and *T*_1/2_ of strychnine and brucine were decreased after pretreatment with TGP. The toxicokinetics study showed that pretreatment with TGP could attenuate the absorption of strychnine and brucine, as well as accelerate their elimination. These results suggest that TGP possesses renoprotective effects.

## 1. Introduction

*Semen Strychni* is a traditional Chinese herb, mainly used to treat rheumatoid arthritis, nervous disease, and traumatic pains and to promote blood circulation and remove blood stasis [1–3]. Total alkaloid from* Semen Strychni* (TAS) is a concentrated bioactive extract of* Semen Strychni*, which mainly consists of strychnine and brucine [4, 5]. Strychnine and brucine possess analgesic, anti-inflammatory, and antitumor effects as well as toxicity [6, 7]. The use of* Semen Strychni* was limited for its extreme toxicity, and it can also cause cardiac arrest, epileptic seizures, and nephrotoxicity [8, 9]. The suggested therapeutic dose of* Semen Strychni* is 0.3–0.6 mg/per day and the dose of its prime toxic ingredient-strychnine is 5–10 mg [10–12]. In previous study,* Semen Strychni* has obvious renal injury [13]. Thus, it is necessary to reduce the nephrotoxicity of* Semen Strychni*.

Some research showed that combining* Semen Strychni* with* Paeonia lactiflora* could reduce the content of strychnine and brucine, increase the therapeutic effect, and reduce the toxicity of* Semen Strychni* [14, 15].* Semen Strychni* and* Paeonia lactiflora *have been compatibly used in many proprietary Chinese medicines such as Gujinwan Capsule, Huoxuezhitong Paste, and Huoluozhenfeng Pill to treat osteopenia and arthralgia and relieve pain. Total glucosides of paeony (TGP) are the predominate effective ingredients of the traditional Chinese herb,* Paeonia alba *radix (roots of* Paeonia lactiflora* Pall.), which mainly contains paeoniflorin and albiflorin. It was reported that TGP possesses analgesic, anti-inflammatory, and immunoregulatory effects [16, 17] and it was exerted renoprotection with antioxidative injury, preventing renal tubulointerstitial injury and blocking TLRs action [18, 19]. It was also reported that TGP plays therapeutic role in experimental diabetic nephropathy [10]; moreover, TGP has already been used to treat chronic nephritis in clinic [20].

As far as we know, few investigation were developed to verify the effects of TGP on nephrotoxicity induced by TAS. The present study was designed to evaluate whether treatment with total glucosides from paeony exerts renoprotective effect against nephrotoxicity induced by total alkaloids from* Semen Strychni.* The levels of serum BUN, Cr, and kidney histopathological changes were used to certify the nephrotoxicity of TAS. What is more, the antidotal effects of TGP on the toxicokinetics of strychnine and brucine in rats were studied.

## 2. Materials and Methods

### 2.1. Material and Regents

**Figure 1:**
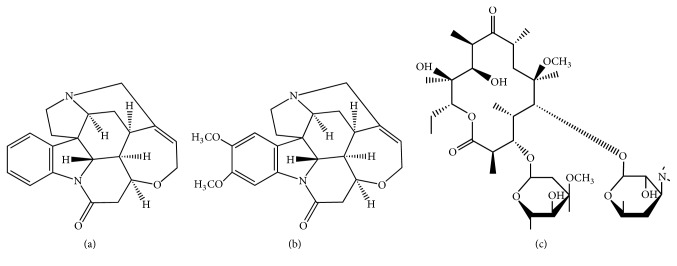
Chemical structures of strychnine (a), brucine (b), and IS clarithromycin (c).

*Semen Strychni*,* Paeonia lactiflora* Pall, and* Aristolochia manshuriensis* (AM) were purchased from Anguo Lengbei Co., Ltd (Hebei, China). All herbs were authenticated by Professor Ying Jia (Pharmacognosy Department, Shenyang Pharmaceutical University). The reference standards of strychnine and brucine were obtained from National Institute for control of Pharmaceutical and Biological Products (Beijing, China). Clarithromycin as internal standard (IS) was provided by Sigma-Aldrich (MO, USA) (Figure 1). The purity of each reference standard was all above 98%. HPLC grade methanol and acetonitrile were purchased from Fisher Scientific (Nanjing, China). HPLC Glacial acetic acid was obtained from Yuwang Industry Co. Ltd. (Shandong, China). Distilled water, prepared from demineralized water, was used throughout experiment.

### 2.2. Herb Preparation

The dried seeds of* Semen Strychni* (100 g) were extracted three times by refluxing with 1 L of 70% ethanol (1 : 10 m/v) for 1 h each time. The collected solvent was concentrated under rotary evaporation to remove all ethanol. The residue was suspended into water and acidified to pH = 2 with HCl (1.0 mol/L) and then extracted with methylene chloride. The pH of aqueous layer was adjusted to 12 with 4.0 mol/L NaOH and then extracted with methylene chloride once more. The methylene chloride layer was concentrated under rotary evaporation to remove all ethanol, and the resultant residue was the TAS extract. The concentrations of strychnine and brucine were determined to be 33.1% and 25.9% by HPLC method.

The powder of* Paeonia lactiflora* Pall radix (250 g) was extracted three times by refluxing with 2 L of 70% ethanol (1 : 8, m/v) for 2 h each time. The filtrates were concentrated by removing all ethanol solvent in rotary evaporation. The residues were suspended into water (0.2 g/mL). The sample solution flowed through glass column (equipped with AB-8 resign). During adsorptive equilibrium, the adsorb-laden column was washed with water firstly and then desorbed with 70% ethanol. The collected solution (70% ethanol) was concentrated by rotary evaporation and the residue was the TGP extract.

Dried stem (100 g) of* Aristolochia manshuriensis* was extracted three times by refluxing with 400 mL of water (1 : 4, m/v) for 2 h each time. The obtained solutions were concentrated by rotary evaporation and the residue was the AM extract.

All extracts were redissolved with carboxymethylcellulose sodium (CMC-Na, 0.5%) and diluted to an appropriate volume.

### 2.3. Animal Treatment

Ninety-six male Sprague-Dawley rats (250 ± 20 g) were kindly supplied by the Experimental Animal Central of Shenyang Pharmaceutical University. All animal-use procedures were in accordance with the regulations for animal experimentation issued by the State Committee of Science and Technology of the People's Republic of China. Animals were housed in laboratory conditions and acclimatized to 7 days before experiments.

Eighty rats were used to access the renal injury induced by TAS and randomly divided into four groups (*n* = 20/group): blank group, positive control group, TGP group, and TAS group. Rats in blank group, positive control group, and TAS group were orally given the approximately same volume of 0.5% CMC-Na solution for 7 days. Rats in TGP group were orally administrated with TGP (280 mg/kg) for 7 days. At the 8th days, rats were orally administrated with 0.5% CMC-Na solution (for the blank group), AM (5.25 g/kg for the positive control group), and TAS (6.5 mg/kg for the TGP and TAS group), respectively.

Blood samples were taken from the abdominal aorta at 0.16, 0.33, 0.5, 1, 4, 9, and 24 h after the final administration on 8th day, and serum was obtained by centrifugation of blood (4,000 rpm for 5 min). Rats (*n* = 2 for each group, 56 in total) were separately sacrificed at 0.16, 0.33, 0.5, 1, 4, 9, and 24 h after the final administration on 8th day, and the kidney tissues were excised and fixed in 10% formalin-saline for at least 24 h and then embedded in paraffin.

Other male SD rats (250 ± 20 g) were used in this part of study. Rats were randomly divided into two groups (*n* = 8), namely, TAS group and TGP group. Before TAS was administrated to rats in the two groups on 8th, TGP group and TAS group were orally given TGP at a dose of 280 mg/kg and 0.5% CMC-Na solution for 7 days, respectively. After oral administration of TAS at a dose of 6.5 mg/kg on 8th day, an aliquot of 300 *μ*L blood samples was taken from the eye ground veins at 0.03, 0.08, 0.16, 0.33, 0.75, 1, 2, 4, 6, and 9 h after oral administration and then centrifuged at 13,000 rpm for 5 min. Plasma samples were collected and stored at −20°C until analysis.

### 2.4. Serum BUN, Cr, and Histopathology

The levels of BUN and Cr in serum were assayed by kits. Kidney samples were sliced into 3 *μ*m wax sections, and the sections were stained with hematoxylin-eosin (H&E) and examined under a light microscope (OLYMPUS, DP72, Japan).

### 2.5. Instruments and LC-MS Conditions

The analysis was performed on a Shimadzu (Japan) LC-MS 2010 EV system equipped with an electrospray ionization (ESI) interface. The liquid chromatographic separation was achieved on CAPCELL PAK C_18_ column (150 mm × 4.5 mm, 5 *μ*m), which was protected by a guard column (4 mm × 2 mm, 5 *μ*m). The mobile phase, consisting of acetonitrile (solvent A) and 0.05% glacial acetic acid water (Solvent B), was delivered at a flow rate of 0.8 mL/min with 25% of the eluent split into the inlet of mass spectrometer. The gradient program was shown as follows: 0-1 min, 13–25% A; 1–3 min, 25% A; 3–5 min, 25–80% A; 5–7 min, 80% A; 7-8 min, 80–13% A; 8–10 min, 13% A. The column and autosampler tray temperature were maintained constantly at 35°C and 4°C, respectively. The inject volume was 10 *μ*L and the run time was 10 min.

The analytes and IS were ionized by ESI source in positive ion mode under the following source conditions: nebulizing gas 1.5 L/min, curved desolvation line (CDL) temperature 250°C, heat block temperature 250°C, detector voltage 1.95 kV, and the other parameters were fixed as tuning file. Selected ion monitoring (SIM) mode was applied for the quantification of [M + H]^+^ at* m/z* 335.05 for strychnine, [M + H]^+^ at* m/z* 395.10 for brucine, and [M + H]^+^ at* m/z* 748.45 for IS, respectively.

### 2.6. Preparation of Calibration Standards and Quality Control Samples

Reference substances of strychnine, brucine, and IS were accurately weighted and dissolved in acetonitrile to get standard stock solutions (200 *μ*g/mL for strychnine, 100 *μ*g/mL for brucine, and 100 *μ*g/mL for IS, resp.). A series of working solutions were prepared by diluting the stock solution with acetonitrile to get the final concentration (5–400 ng/mL for strychnine, 5–100 ng/mL for brucine, and 1000 ng/mL for IS). Quality control (QC) solutions in plasma were prepared in the same way at low, middle, and high concentrations: 15, 100, and 320 ng/mL for strychnine; and 15, 50, and 80 ng/mL for brucine. All solutions were stocked at 4°C.

### 2.7. Plasma Sample Preparation

An aliquot of 100 *μ*L plasma was spiked with 20 *μ*L of IS, followed by adding 300 *μ*L acetonitrile. The mixed solutions were vortexed for 3 min and centrifuged at 13,000 rpm for 5 min; then the supernatant was transferred into a clean centrifuge tube and dried under a stream of nitrogen. The residue was reconstituted with 100 *μ*L of initial mobile phase and vortexing for 1 min, followed by centrifuging for another 10 min. Finally, an aliquot of 10 *μ*L of the supernatant was injected into the HPLC-MS system for analysis.

### 2.8. HPLC-MS Method Validation

In our study, the HPLC-MS method was validated in accordance with FDA guidelines [21, 22], including selectivity, linearity, lower limited of quantification (LLOQ), precision, accuracy, extraction recovery, matrix effect, carry-over, and stability.

Selectivity was accessed by comparing chromatograms of blank plasmas obtained from six different SD rats, blank plasma sample spiked with analytes and IS, and plasma obtained 0.33 h after oral administrated of TAS.

The linearity of the assay was assessed by analyzing the calibration curves using least-squares linear regression of the peak area of the analytes to IS versus the nominal concentration of the calibration standard with a weighted factor (1/*C*^2^). The LLOQ was defined as the lowest concentration on calibration curve with an acceptable accuracy (between 80 and 120%) and the precision below 20%.

Accuracy and precision (inter- and intraday) were measured by determining QC samples at three concentrations levels with 6 replicates on three consecutive validation days. Accuracy was evaluated as relative error (RE%) and precision was evaluated as relative standard deviation (RSD%).

Recovery was calculated by comparing peak areas from extracted samples with those postextracted samples spiked with analytes and IS (*A*). Matrix effect was calculated by calculating the peak area ratios of *A* to pure standard solution containing equivalent amounts of the compounds. Both were performed at three QC levels.

Stability studies in plasma samples were also conducted at three QC levels in several different storage conditions: at room temperature for 12 h, at −20°C for at least 15 d, after three freeze-thaw cycles, and 12 h after preparing at 4°C.

Carry-over was assessed by injecting two blank sample extracts after upper limit quantification (ULOQ), and peak area found in first blank sample should be less than 20% of the peak area at LLOQ of analytes.

### 2.9. Statistical Analysis

The concentration of the analytes was calculated by the corresponding calibration curve. Toxicokinetics studies were processed by DAS 2.1.1 software package (Chinese Pharmacological Society) to calculate half-life (*T*_1/2_), the maximum plasma concentration (*C*_max_), the corresponding time (*T*_max_), and the area under concentration-time curve (AUC). Results of renal function markets and the toxicokinetics parameters were expressed as mean ± standard deviation (mean ± SD). Statistical analysis was performed using SPSS 19.0 software package by one-way analysis of variance (ANOVA) followed by Tukey-Kramer multiple comparison tests. Statistical significance was considered when the value of *p* < 0.05.

## 3. Results and Discussion

### 3.1. Assessment of Renal Function Markers and Analysis

**Figure 2:**
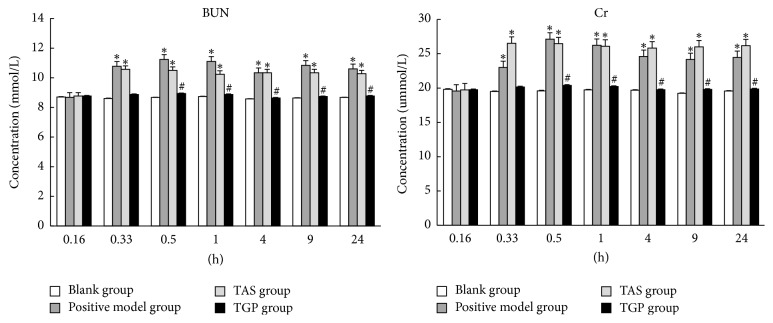
The levels of serum BUN and Cr of four groups on different time points. All values are indicated as mean ± SD (*n* = 8). ^*∗*^*p* < 0.05 significant difference between other groups and blank group. ^#^*p* < 0.05 significant difference between TGP group and TAS group.

 Experimental and clinical studies have demonstrated that renal damage usually is accompanied by serum biochemical changes. For example, patients with acute renal failure usually have higher BUN and Cr [23, 24]. Increased level of serum BUN indicated the weaker ability of glomerular filtration rate and the loss of renal tubular reabsorption selectivity in acute and chronic nephritis. Moreover, increased level of serum Cr indicates that renal excretion has been severely damaged [25, 26]. The results of serum BUN and Cr in this study are presented in Figure 2. In comparison with the blank group, levels of BUN and Cr in serum were significantly increased (*p* < 0.05) after the final administration on 8th day from 0.16 h to 24 h in the TAS and positive control group, which indicated that kidney was damaged. The levels of serum BUN and Cr in TGP group showed no significant difference comparing to blank group, but remarkably decreased (*p* < 0.05) from 0.16 h to 24 h comparing to TAS group. This result suggested that TGP could attenuate the nephrotoxicity caused by TAS.

### 3.2. Histopathology

**Figure 3:**
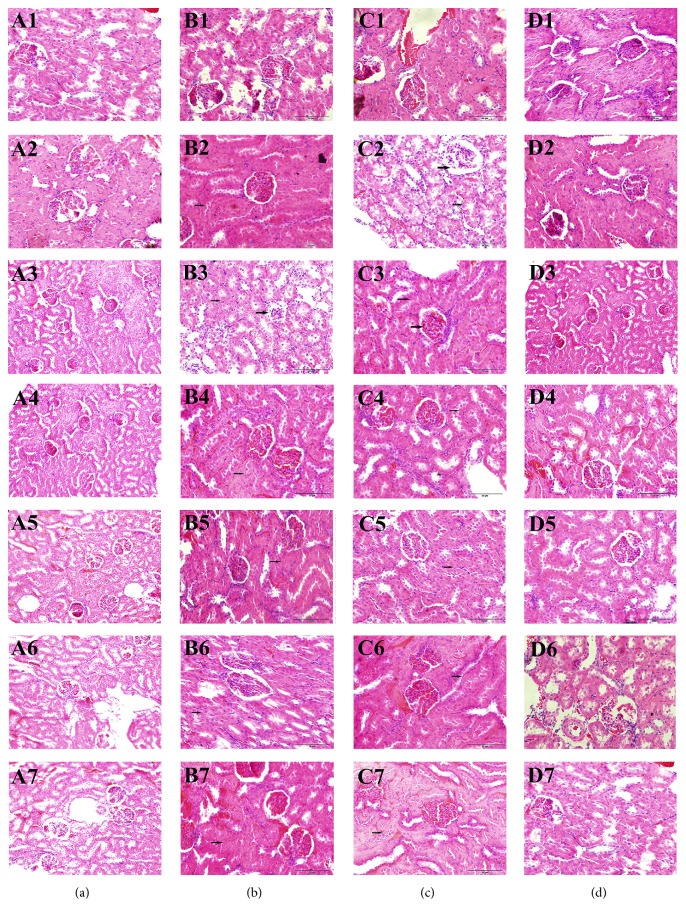
Representative histopathological photographs of SD rats' kidney sections (×100) in different groups. Rats in blank group (a), rats in positive control group (b), rats in TAS group (c), rats in TGP group (d). 1–7: kidney condition at 0.16, 0.33, 0.5, 1, 4, 9, and 12 h (different kinds of pathology lesions were marked by different arrows).

Histopathological changes were examined to investigate TAS-induced nephrotoxicity, which can directly help us understand the histopathological changes in kidney. Histopathological changes in the rat kidney are presented in Figure 3. Figure 3 (A1–A7) shows no abnormal morphological changes in the kidney specimen in the rats of blank group. Rats in positive group (Figure 3, B2–B7) and TAS group (Figure 3, C2–C7) show that the kidney sections gradually presented tubular epithelium cloudy swelling, degeneration, and glomerular atrophy. No abnormal morphological changes appeared in TGP group rats (Figure 3, D1–D7). Histopathology study demonstrated that TGP could protect kidney against TAS-induced renal injury, which is supported by the results of renal function biomarkers. The reasons of renal function markers and histopathology changes might be explained as TGP could inhibit macrophage infiltration into kidney, resist renal inflammation, and renal tubular epithelial cell transdifferentiation and exert antioxidative stress effect [18, 27]. The results of renal function biomarkers and histopathology changes suggested that TGP might display renoprotective effects on nephrotoxicity induced by TAS.

### 3.3. HPLC-MS Method Validation

**Figure 4:**
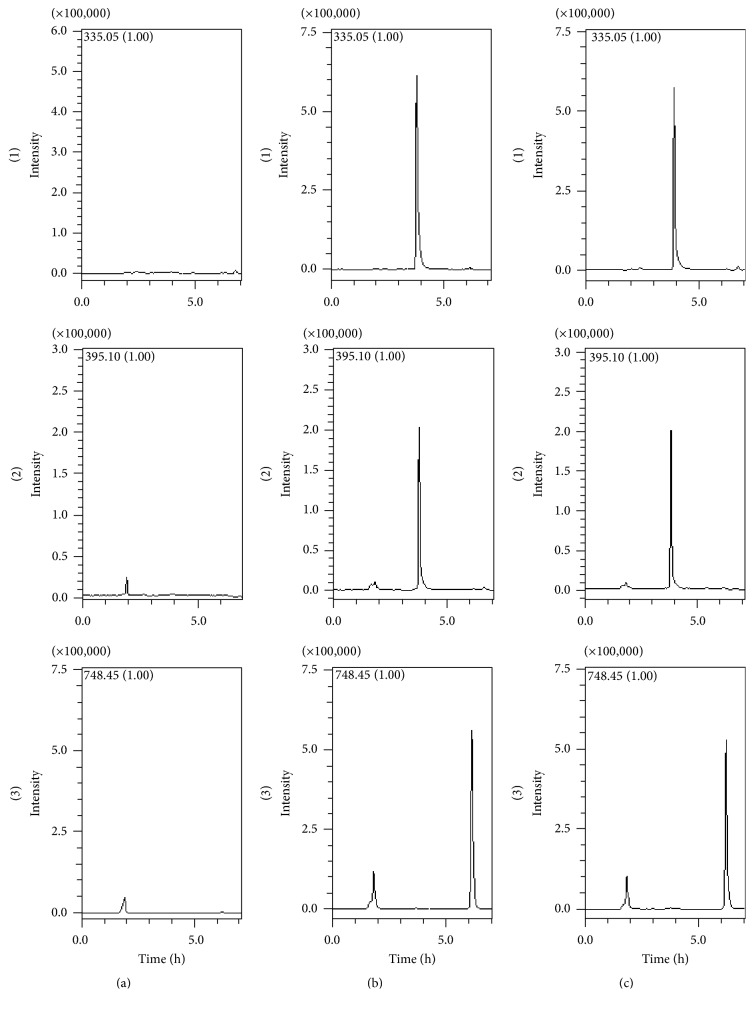
Typical chromatograms of strychnine (1), brucine (2), and IS (3). (a) Blank plasma, (b) blank plasma with strychnine, brucine, and IS, and (c) plasma sample obtained 0.33 h after oral administration of TAS extract.

No interfering peak from endogenous substances was observed in the representative chromatogram of blank plasma sample at the retention time of the analytes and IS. The retention times of strychnine, brucine, and IS were 3.82, 3.90, and 6.20 min, respectively (Figure 4). The calibration curves showed good linearity in the range of 5–400 ng/mL for strychnine and 5–100 ng/mL for brucine. Typical regression equations of the calibration curves were *y* = 0.0180*x* + 0.0093 (*r* = 0.9961) for strychnine and *y* = 0.0120*x* + 0.0062 (*r* = 0.9987) for brucine. The LLOQs of strychnine and brucine were 5 ng/mL with the RSD% less than 10.2%, which was within acceptable limits. Precision and accuracy were satisfactory at three levels of QC samples. The extraction solvent used in experiment showed good extraction recovery. The recoveries of strychnine and brucine in three QC concentrations were 81.1–91.9% and 82.2–92.2%, and the recovery of IS was 95.5%. The results of matrix effect were 88.3–93.4% for strychnine and 87.5–104.6% for brucine, and the matrix effect of IS was 98.9%. The analytes were stable (RE% within 11.4% and RSD% below 9.1%) under tested conditions and no carry-over was found for the method (showed in Table 1). The results indicated that the developed method was reliable and reproducible to quantitate strychnine and brucine in rat plasma.

### 3.4. Toxicokinetics Results

**Figure 5:**
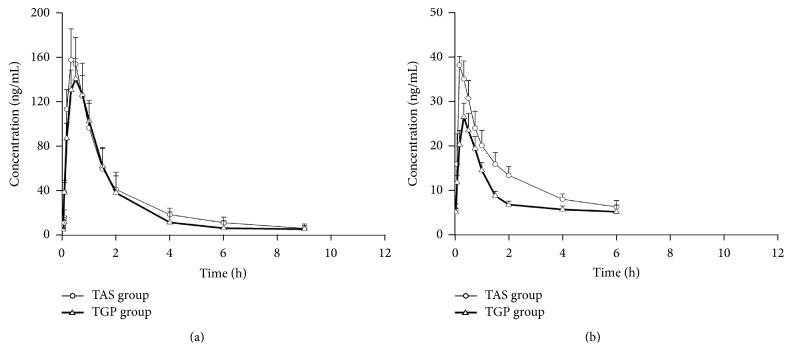
Mean plasma concentration-time curves of strychnine (a) and brucine (b) in rat plasma after oral administration of TAS extract at a dose of 6.5 mg/kg (*n* = 8).

The validation method was successfully applied to the toxicokinetics of strychnine and brucine in rat plasma after oral administration of TAS. The concentration-time profiles of TAS group and TGP group are illustrated in Figure 5, and the corresponding toxicokinetics parameters are summarized in Table 2.

Oral administration of TAS in rat can cause obvious nephrotoxicity, which is supported by biomarkers and pathological tests. The dose of TAS was verified according to previous study and based on our preliminary experiments [3]. AM was selected as nephrotoxic positive control herb for obvious nephrotoxicity [28]. Moreover, the dose of TGP was selected referring to the reports on TGP preventing renal damage in diabetic rats [18].

As is shown in Table 2, there were statistical differences in several toxicokinetics parameters including *T*_1/2_, *T*_max_, *C*_max_, and AUC for analytes between TAS group and TGP group. Comparing with TAS group, *T*_1/2_ of strychnine and brucine in TGP group significantly decreased (*p* < 0.05) and their *T*_max_ postponed (*p* < 0.05). The parameters of *C*_max_ and AUC decreased (*p* < 0.05) significantly in brucine and showed slight decrease (*p* > 0.05) in strychnine in TGP groups comparing to TAS group. These changes indicated that pretreatment of TGP could affect the toxicokinetic profiles of strychnine and brucine and attenuate the toxicity of TAS.

The toxicity of TAS was does-depended, and the decreased *C*_max_ and AUC in TGP group might contribute to preventing strychnine and brucine from the lethal does. Their increased *T*_max_ were also observed in TGP group, which may be caused by activating P-gp property of paeoniflorin [29]. The increase of *T*_max_ indicated that the delay of absorption of strychnine and brucine might contribute to the renoprotective effect of TGP.

In TGP group, the decreased *T*_1/2_ of strychnine and brucine were observed, illustrating that TGP may have accelerated their elimination. Some researchers proved that strychnine and brucine are substrates of CYPs [30, 31]. Furthermore, paeoniflorin and albiflorin (main effective compounds of TGP) were reported to regulate the activity of CYP3A4 and CYP2D6 with varying degrees [32]. It illuminated that TGP induced the activity of CYP3A4 and CYP2D6, accelerated the metabolism of strychnine and brucine, and thus reduced the toxicity of TAS. It might be another proof to explain the decreased *T*_1/2_ of strychnine and brucine and the renoprotective effect of TGP.

The preventive and therapeutic capacity of TGP against renal damage associated with anti-inflammatory and antioxidant effects might be another reason for TGP ameliorating nephrotoxicity induced by TAS [33, 34]. All those results above indicated that TGP could protect the kidney against nephrotoxicity induced by TAS and attenuate the toxicity of* Semen Strychni*. This study supplied a guidance for the safe medication of* Semen Strychni* in clinic and was utilized in clinic for protecting kidney against nephrotoxicity induced by not only TAS but also other drugs.

## 4. Conclusions

In conclusion, this study indicated that pretreatment with TGP could recover the levels of BUN and Cr, ameliorate kidney morphological changes, and thus exert renoprotective effect against nephrotoxicity induced by TAS. The toxicokinetics results of two groups showed that pretreatment with TGP could attenuate the absorption of strychnine and brucine and accelerate their elimination and may demonstrate the possible mechanism of TGP against nephrotoxicity induced by* Semen Strychni*. What is more, this study might be further utilized in clinic for nephrotoxicity induced by other drugs.

## Figures and Tables

**Table 1:** Precision, accuracy, recovery, and matrix effect of HPLC-MS method for analysis of analytes and IS (*n* = 8).

Analytes	*C* (ng/mL)	Intraday	Interday	Accuracy	Recovery	Matrix effect
		(RSD%)	(RSD%)	(RE%)	(%, mean ± SD)	(%, mean ± SD)
Strychnine	15	13.0	10.3	−15.4	81.1 ± 5.6	92.5 ± 6.8
	140	7.5	7.9	9.1	95.4 ± 4.3	93.4 ± 6.4
	300	4.6	6.5	4.8	91.9 ± 5.3	88.3 ± 5.6
Brucine	15	11.8	10.0	−14.7	82.2 ± 12.6	87.5 ± 6.4
	50	6.1	5.3	6.1	92.2 ± 4.7	104.6 ± 5.2
	80	3.6	4.9	3.5	91.8 ± 8.2	99.3 ± 4.2

**Table 2:** Toxicokinetics parameters of strychnine and brucine in rat plasma after oral administration of TAS extract (mean ± SD, *n* = 8).

Parameters	Strychnine		Brucine	
	TAS group	TGP group	TAS group	TGP group
*T*_1/2_ (h)	2.5 ± 0.5	1.4 ± 0.3^*∗*^	2.7 ± 0.4	1.1 ± 0.2^*∗*^
*T*_max_ (h)	0.3 ± 0.2	0.5 ± 0.1^*∗*^	0.2 ± 0.1	0.3 ± 0.2^*∗*^
*C*_max_ (ng/mL)	172.6 ± 28.2	153.1 ± 16.3	40.5 ± 2.8	28.1 ± 4.3^*∗*^
AUC_0−*t*_ (ng h/mL)	308.9 ± 76.2	258.8 ± 53.8	79.2 ± 8.1	42.0 ± 5.0^*∗*^
AUC_0−*∞*_ (ng h/mL)	330.1 ± 80.8	275.6 ± 64.9	99.7 ± 13.9	45.1 ± 5.6^*∗*^

^*∗*^ *p* < 0.05 significant differences between TAS group and TGP group.
